# Research advances in cryopreserved preparations of mesenchymal stem cells: technical innovations, application challenges, and quality control

**DOI:** 10.3389/fbioe.2026.1717539

**Published:** 2026-01-21

**Authors:** Yuhao Wang, Kexin Yang, Shengmeng Yuan, Fangjun Huo, Chao Yang, Weidong Tian

**Affiliations:** 1 State Key Laboratory of Oral Diseases and National Clinical Research Center for Oral Diseases and Engineering Research Center of Oral Translational Medicine, Ministry of Education and National Engineering Laboratory for Oral Regenerative Medicine, West China Hospital of Stomatology, Sichuan University, Chengdu, Sichuan, China; 2 Department of Oral and Maxillofacial Surgery, West China Hospital of Stomatology, Sichuan University, Chengdu, Sichuan, China; 3 Chengdu Shiliankangjian Biotechnology Co., Ltd., Chengdu, Sichuan, China; 4 Sichuan Tianfu Cell Quality Detection and Evaluation Center Co., Ltd., Chengdu, Sichuan, China

**Keywords:** “off-the-shelf” therapy, cryopreservation, mesenchymal stem cells (MSCs), potency assay, quality control, regenerative medicine

## Abstract

Although mesenchymal stem cells (MSCs) are among the most promising cell types for regenerative medicine, the lack of mature “off-the-shelf” cryopreserved preparations limits their widespread clinical application. This represents a critical bottleneck and an often-underestimated complication of the cryopreservation process, which leads not only to significant reduction in viable cell yield but also to subtle yet consequential perturbations in therapeutic function. This review distinguishes itself by critically synthesizing recent advances through the lens of the integrated “vial-to-vein” pathway, emphasizing how cryopreservation-induced attrition of functional potency—particularly in immunomodulation and paracrine signaling—compromises clinical efficacy. We systematically analyze the evolution beyond conventional dimethyl sulfoxide (DMSO)-based media towards next-generation, bioinspired cryoprotectants and storage strategies designed to safeguard these critical biological attributes. We then review the cryopreservation effects on MSCs morphology, surface marker consistency, and multipotent differentiation as well as their fundamental immunomodulation. Subsequently, the review consider the efficiency of cryopreserved MSCs in different disease models like cardiovascular diseases — respiratory diseases and chronic kidney disease. Finally, we discuss the pivotal transition in quality control, arguing for a multi-pillar paradigm that integrates precise molecular identity testing with clinically relevant functional potency assays tailored to specific indications. Crucial in the pursuit of this integrated understanding is to ensure a set of consistent, reliable and coherent properties by which next-generation MSCs therapies can be evaluated. Yet correlating these *in vitro* metrics with clinical efficacy remains the single greatest hurdle.

## 1 Introduction

For a long time, MSCs have been central to cell therapy research (Hansen et al., 2022; Margiana et al., 2022). They are multipotent and can be gathered from tissues like bone marrow and fat. Furthermore, they are capable of forming bone, cartilage, and fat cells (Naji et al., 2019). Initially, it was believed that these cells repaired the body simply by replacing damaged tissue; however, this model is now outdated. Many studies have found that MSCs are effective even without long-term engraftment in the body (Spees et al., 2016), indicating that their mechanism of action is more complex.

Today, we see MSCs differently (Caplan, 2017). They are now considered “medicinal signaling cells” that act primarily through paracrine signals (Margiana et al., 2022). Instead of replacing tissue, MSCs release various factors such as cytokines and growth factors, which alter the local body environment (Spees et al., 2016). These secreted molecules have powerful effects. They can help cells survive, promote new blood vessel growth, reduce scarring, and, most importantly, regulate the immune system (Naji et al., 2019; Spees et al., 2016). For example, MSCs exert immunomodulatory effects through multiple mechanisms: they can inhibit the activation and proliferation of T-cells, induce macrophages to polarize toward the anti-inflammatory M2 phenotype, and modulate the function and differentiation of B-cells and dendritic cells. This makes them a promising treatment for many inflammatory diseases and for Graft-versus-Host Disease (GVHD) (Hansen et al., 2022; Naji et al., 2019).

Bringing MSCs from the lab to the clinic as a therapeutic product creates significant logistical challenges. Freshly cultured cells do not last long, are expensive, and hard to standardize for widespread use (Crow, 2019). This is why cryopreservation is so critical. It permits the creation of “off-the-shelf” allogeneic MSC therapies. Importantly, large and consistent batches can be produced. These products can then be stored for long periods and used as needed, offering a drug-like model of administration (Crow, 2019). This model could lower costs and make these therapies more accessible.

But there is a catch. The very process of freezing and thawing is harsh on the cells. It can damage them, affecting not just their viability but also their crucial biological functions. This leads to the central question of this review: are cryopreserved MSCs as effective as fresh ones? We are particularly concerned about their immunomodulatory capacity, which is key to their clinical success. Simple viability tests after thawing do not tell the whole story and often do not correlate with therapeutic potency (Hansen et al., 2022). Therefore, given the variability between donors and manufacturing processes, better functional assays are needed to ensure product quality (Hansen et al., 2022; Crow, 2019).

**FIGURE 1:**
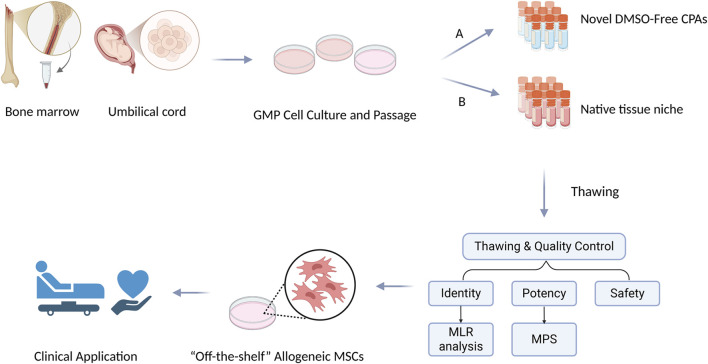
Workflow for creating an “off-the-shelf” MSCs therapy. MSCs are isolated from tissues like bone marrow or umbilical cord and expanded under GMP. A key step is cryopreservation, shown here with two advanced methods: **(A)** using novel DMSO-free CPAs like trehalose-based polymers, and **(B)** preserving MSCs in their native tissue niche. After thawing, cells must pass several critical quality control (QC) tests. These confirm the cells’ identity (e.g., via transcriptomic signature), ensure their safety (free of contaminants), and measure their potency for the specific therapy, using assays like MLR for immunomodulation or MPS for vasculogenesis.

To address these issues, this review will systematically examine how cryopreservation truly impacts MSCs. We will look at the latest technologies, the effects on cell biology (like morphology and differentiation), and the performance in various disease models. We will also cover the essential manufacturing and regulatory steps needed to create standardized, cryopreserved MSCs products, as outlined in Figure 1.

## 2 Fundamentals and development of MSCs cryopreservation technology

**FIGURE 2:**
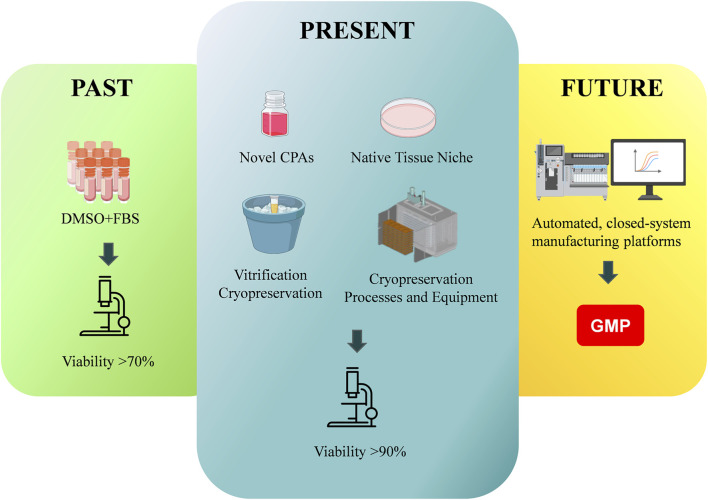
The evolving paradigm of MSC cryopreservation: from traditional DMSO + FBS-based cryoprotectants to novel cryoprotective agents, native tissue niches, and vitrification cryopreservation, extending toward promising horizons such as automated manufacturing platform.

How can we create a successful “off-the-shelf” MSCs therapy? The answer starts with reliable cryopreservation methods that preserve cell viability and function. Here, we examine the fundamental science of freezing cells, tracing the evolution from traditional DMSO + fetal bovine serum (FBS)-based cryoprotectants to novel cryoprotective agents, native tissue niches, and vitrification cryopreservation, extending toward promising horizons such as automated manufacturing platform (Figure 2).

### 2.1 Traditional cryopreservation media: composition and mechanisms

**FIGURE 3:**
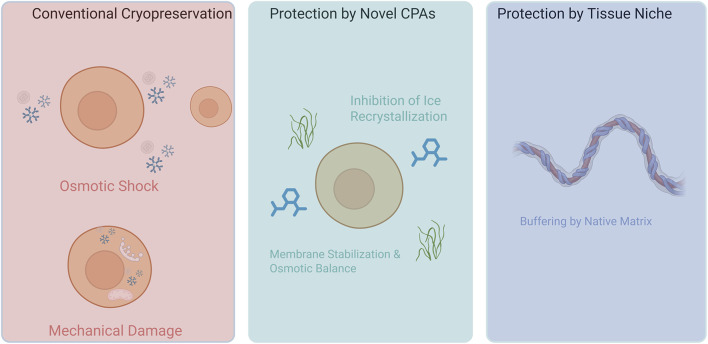
Mechanisms of cryoinjury in MSCs and a comparison of advanced protective strategies. This figure illustrates three key concepts in MSCs cryopreservation. On the left, the process of conventional cryopreservation is shown, where the use of agents like DMSO can still lead to significant cell damage from extracellular ice crystal formation (causing osmotic stress) and intracellular ice formation (causing mechanical rupture), in addition to direct cytotoxicity. In the center, the protective mechanism of novel, advanced CPAs like trehalose-based or zwitterionic polymers is depicted. These agents mitigate cryoinjury by inhibiting ice recrystallization (IRI), stabilizing the cell membrane, and balancing osmotic shifts without inherent toxicity. On the right, the concept of preserving MSCs within their native tissue niche is shown. The natural extracellular matrix (ECM) acts as a protective scaffold, physically buffering against stresses and maintaining the cellular microenvironment, which enhances post-thaw viability and recovery.

When cells are frozen, they are pushed to their physical limits. The formation of ice crystals and sudden changes in osmotic pressure are the two main culprits of irreversible damage (Figure 3). As the external medium cools, extracellular ice forms first. This causes solute concentration and creates a hypertonic environment. Consequently, water flows out of the cells, resulting in osmotic shock and dehydration. Conversely, rapid cooling can lead to lethal intracellular ice formation (IIF), physically rupturing membranes and organelles. This aligns with Mazur’s “two-factor hypothesis,” which states that both excessively slow and excessively fast cooling are lethal (Bojic et al., 2021). Moreover, during warming, ice recrystallization and devitrification impose additional mechanical damage. On a biochemical level, freeze–thaw processes may induce protein denaturation, mitochondrial dysfunction, reactive oxygen species generation, and premature activation of programmed cell death pathways (e.g., apoptosis and necrosis)—cumulatively determining post-thaw viability and function (Chang and Zhao, 2021; Li et al., 2022).

A comparative study on MSCs revealed that murine MSCs (mMSCs) showed a post-thaw viability of 91.5% ± 5.6%, whereas human MSCs (hMSCs) achieved only 82.9% ± 4.3%. This disparity is attributed to differences in cell volume and surface-area-to-volume ratios (SA/V): mMSCs exhibit an SA/V of approximately 0.65 μm^-1^, compared to ∼0.34 μm^-1^ for hMSCs. The higher SA/V facilitates more efficient dehydration, thereby reducing susceptibility to IIF—emphasizing the need for tailored cooling rates according to cell type, consistent with fundamental cryobiological principles (Jang et al., 2017; Capicciotti et al., 2015; Yi et al., 2014; Prickett et al., 2015). Importantly, such biophysical variations also exist between MSCs from different human tissue sources, which underlies their distinct cryopreservation vulnerabilities. For instance, adipose-derived MSCs (AD-MSCs) typically have a larger cell volume and lower SA/V ratio compared to the smaller, more spindle-shaped bone marrow MSCs (BM-MSCs). This fundamental difference in cellular geometry makes AD-MSCs theoretically more susceptible to intracellular ice formation during rapid cooling, necessitating tailored optimization of cooling rates for each cell type.

The classic cryopreservation of MSCs has long relied on a simple formula utilizing two key agents: DMSO to prevent intracellular ice formation (Margiana et al., 2022; Naji et al., 2019) and FBS to provide protein stability and protect cell membranes (Svoradová et al., 2023; Gao et al., 2021; Duarte Rojas et al., 2024). Yet, this approach is far from ideal. DMSO is known to be toxic to cells (Spees et al., 2016; Crow, 2019). FBS presents a host of other challenges for clinical translation, including inconsistent performance between batches and the risk of immune reactions (Duarte Rojas et al., 2024). The search for better options was therefore inevitable. The adoption of human platelet lysate (HPL) as a safer alternative to FBS is a prime example of this shift (Svoradová et al., 2023; Duarte Rojas et al., 2024), driven by the fundamental weaknesses of the original method.

### 2.2 Optimization strategies for cryopreservation media

Researchers have taken two main approaches to address the limitations of the classic DMSO + FBS medium. The first approach is conservative: improving the existing formula by adding new ingredients, often referred to as “cryoprotective adjuvants.” Examples include polyethylene glycol (PEG), which physically protects the cell membrane (AlHindi and Philip, 2021), and non-penetrating sugars such as trehalose, which shield cells from extracellular ice damage (Gao et al., 2021; Andreoli et al., 2024). To solve the FBS problem, a defined protein like bovine serum albumin (BSA),which is derived from bovine sources, can be used instead for stability (Duarte Rojas et al., 2024).

However, the inclusion of animal serum may not be necessary for clinically applicable cells, mainly because of the risks of xenogenic viral transmission (e.g., prions) and variability in serum composition, sources, and production lots affecting cultured cell phenotypes (Cimino et al., 2017; Guiotto et al., 2020). A more radical approach, however, aims to completely remove the problematic ingredients. This has led to the design of fully serum-free and xeno-free systems. By replacing FBS and sometimes DMSO with synthetic, less toxic components, these formulations offer a much safer and more consistent product for clinical use. For instance, formulations using glycerol and specific amino acids like isoleucine have demonstrated good post-thaw survival and functional recovery of MSCs with reduced immunogenicity compared to DMSO-based protocols (de Camargo et al., 2025; Larsen et al., 2024). Studies indicate that glycerol provides cryoprotection by osmotically shielding cells from ice crystal damage, while amino acids such as proline act by preventing intracellular crowding and ectoine functions as an osmoprotectant against hypertonic stress. The clinical-scale feasibility of such xenofree strategies is further supported by advances in serum-free culture systems, exemplified by Lonza’s commercially available medium designed for expanding umbilical cord-derived MSCs while maintaining their critical biological properties (Wu et al., 2014). Using these cryoprotectants, either individually or in combination, a methylcellulose-based cryomedium was developed to replace DMSO/serum-containing solutions effectively. Results demonstrated 99% post-thaw viability with optimized formulations. Notably, a composite solution containing 1% proline and 10% ectoine achieved 90% viability (Freimark et al., 2011). The development of novel, polymer-based cryoprotective agents (CPAs)represents the next frontier, offering a move away from traditional small-molecule protectants altogether.

### 2.3 Cryopreservation via native tissue niche

The native tissue niche is an intrinsic three-dimensional microenvironment within organisms. It consists of tissue-specific ECM, signaling molecules, and adjacent cells. This architecture provides physical and biochemical cues essential for stem cell survival, self-renewal, and differentiation (Yang et al., 2016). Wharton’s jelly—the gelatinous matrix within umbilical cord tissue—features an ECM that forms a natural 3D scaffold. Research confirms this structure, enriched with collagens, hyaluronic acid, and other components, effectively mimics the *in vivo* stem cell niche by providing MSCs with adhesion sites and mechanical support. Remarkably, cryopreserved MSCs in processed umbilical cord tissue maintain expression of stemness markers (CD73/CD90/CD105) and multilineage differentiation potential. Experimental evidence demonstrates that post-thaw umbilical MSCs retain high efficiency in differentiating into osteoblasts, chondrocytes, and adipocytes. This validates the 3D niche’s role in preserving cellular functional stability (Ding et al., 2011; Seo et al., 2021). Within dental pulp niches, neurovascular networks deliver stem cell factor (SCF) and maintain hypoxic conditions that preserve dental MSCs quiescence (Sharpe, 2016). Therefore, native tissue niches serve as protective scaffolds that mechanically buffer external stresses and sustain cellular microenvironments, thereby enhancing post-thaw survival rates and functional recovery.

In practice, the cryopreservation of intact tissue niches generally requires the addition of CPAs to mitigate intracellular ice formation and osmotic stress during freezing and thawing. Both permeating CPAs (e.g., DMSO, ethylene glycol) and non-permeating CPAs (e.g., trehalose, sucrose, hydroxyethyl starch) have been employed to improve cell recovery within the preserved ECM. Importantly, the ECM itself does not usually require enzymatic removal or disruption prior to cryopreservation; rather, its intact structure functions synergistically with CPAs to protect embedded cells by reducing ice crystal propagation and maintaining local osmotic balance. Recent studies suggest that combining native ECM scaffolds with optimized CPAs cocktails achieves superior post-thaw viability and functionality compared with isolated cell suspensions, underscoring the complementary roles of chemical protection and structural microenvironments in tissue-level cryopreservation (Seo et al., 2021; Whaley et al., 2021). A comparative analysis of various cryopreservation media is listed in Table 1.

**TABLE 1:** Comparison of GMP-grade cryopreservation media for MSCs and future improvement opportunities.

Core strategy	Representative composition	Advantages	Limitations	Future improvement opportunities	Reference
Traditional DMSO/Serum-Based	5%–10% DMSO + Fetal Bovine Serum (FBS)	• Well-established, widely used protocol • Efficacy validated for various cell types • Typically ensures high post-thaw viability	• DMSO cytotoxicity (impairs cell function, infusion-related reactions) • Xenogenic risks of FBS (immunogenicity, pathogen transmission, batch-to-batch variability) • Non-compliant with strict “xeno-free” clinical manufacturing	• Develop novel biocompatible CPAs to reduce/replace DMSO. E.g., zwitterionic polymers that stabilize cell membranes and inhibit ice crystallization offer a path to DMSO-free formulations • Completely replace FBS with clinical-grade alternatives like HPL, optimizing pathogen inactivation and batch consistency processes	Gao et al. (2021), Nishigaki et al. (2011), Kaushal et al. (2022)
DMSO/Human Platelet Lysate (HPL)-Based	5%–10% DMSO + Human Platelet Lysate (HPL)	• Xeno-free, higher clinical safety • HPL provides rich growth factors and adhesion proteins, aiding post-thaw recovery • A mainstream clinical-grade alternative to FBS.	• Batch-to-batch variability persists with HPL • DMSO toxicity remains a concern • Requires rigorous HPL donor screening and pathogen inactivation	• Develop chemically defined HPL mimetics or recombinant protein formulations to fundamentally address batch variability • Combine HPL with novel non-penetrating CPAs (e.g., trehalose derivatives) to aim for DMSO concentrations below 5%, reducing toxicity while maintaining high cell recovery	Yamatoya et al. (2023), Murray et al. (2024)
Serum-Free/Chemically Defined	Low DMSO (or Glycerol) + Recombinant Proteins (e.g., rAlbumin) + Specific Amino Acids/Sugars	• Chemically defined, xeno-free, high batch-to-batch consistency • Clear regulatory path, high safety • Eliminates unknowns associated with serum	• Complex and costly formulation development • Cryoprotection for some MSC sources may be inferior to serum-containing media • Complete DMSO removal remains challenging	• Rational design of multifunctional synthetic polymers as core CPAs. E.g., trehalose-based polyethers with Ice Recrystallization Inhibition (IRI) activity can provide excellent protection independent of DMSO • Leverage high-throughput screening platforms to rapidly optimize serum-free, DMSO-free formulations for different tissue-specific MSCs	Strecanska et al. (2025), Wang et al. (2022a)
DMSO-Free/Novel CPA-Based	Ethylene Glycol/1,2-Propanediol + Sucrose/Trehalose + Polymers (e.g., PVA)	• Avoids DMSO toxicity and related adverse effects entirely • Provides an option for DMSO-sensitive patient groups • Represents a cutting-edge technological direction	• Formulations can be complex, requiring fine-tuning of osmotic stress and toxicity balance • Clinical safety and efficacy data for some novel CPAs are still limited • Manufacturing and QC standards need further development	• Explore intracellular delivery technologies to efficiently introduce non-penetrating CPAs like trehalose into the cytosol, enabling intracellular protection and independence from traditional permeable CPAs • Combine novel CPAs with physical protection strategies (e.g., hydrogel encapsulation) for a synergistic effect, enhancing cryopreservation outcomes while reducing total CPA concentration	Gao et al. (2021), Li et al. (2025)
Bioinspired/Native Niche	Intact or Engineered Extracellular Matrix (ECM) + Low CPA Concentration	• Mimics the in vivo stem cell niche, better preserving cellular function (e.g., immunomodulation, differentiation) • ECM acts as a physical barrier, mitigating mechanical ice damage • Potentially reduces the requirement for high CPA concentrations	• Standardization and scalable production of tissue-derived ECM is challenging • More complex freezing/thawing processes, difficult to integrate into existing pipelines • Presents new regulatory challenges	• Apply synthetic biology and material engineering to create bioinspired ECM hydrogels. These materials provide controllable physico-chemical cues and act as 3D protective scaffolds during freezing, significantly enhancing functional cell recovery • Establish standardized “organoid” cryopreservation protocols for 3D microtissues and develop corresponding inline monitoring technologies to facilitate clinical translation	Ortiz Silva et al. (2024), Pu et al. (2025)

### 2.4 Vitrification cryopreservation

Vitrification cryopreservation employs rapid cooling of highly concentrated cryoprotective agents (CPAs) in liquid nitrogen to achieve a glass-like amorphous solid state, effectively inhibiting ice crystal formation and thereby preserving cellular structural integrity and function (Fahy, 1986; Jomha et al., 2012; Rall and Fahy, 1985; Vajta et al., 1998; Dias et al., 2023). A typical protocol involves two sequential steps: initial equilibration with 20% (v/v) ethylene glycol at room temperature for 5 min, followed by direct immersion into a cryomedium containing 40% (v/v) ethylene glycol, 0.3 mol/L sucrose, 18% (w/v) ficoll and 20% fetal bovine serum in liquid nitrogen. Although this conventional formulation achieves high post-thaw viability, its clinical applicability is severely limited by several factors. The high concentrations of CPAs, particularly ethylene glycol, induce significant cytotoxicity and osmotic stress, compounded by risks associated with residual CPA exposure post-thaw (Han et al., 2023). Furthermore, the inclusion of FBS introduces xenogeneic components, posing immunogenicity risks, potential pathogen transmission, and substantial regulatory hurdles for clinical translation (Lawson et al., 2011). Consequently, research has shifted toward developing safer, xeno-free formulations. For instance, a DMSO-free cocktail comprising ethylene glycol, 1,2-propanediol, sucrose, and polyvinyl alcohol (PVA) as an additive has been validated for vitrification of umbilical cord blood-derived MSCs (UCB-MSCs). Comparative analyses demonstrate that this approach significantly outperforms programmed freezing; PVA supplementation elevated post-thaw viability from 71.2% to 95.4% (p < 0.01), whereas conventional programmed freezing yielded suboptimal survival (<45%) (Wang et al., 2011). Nevertheless, even improved formulations raise concerns regarding osmotic stress, potential cytotoxicity from high concentrations of alternative CPAs like EG or 1,2-propanediol, and the effects of residual additives. Therefore, while avoiding DMSO and serum marks a critical advance toward clinical translatability, further optimization of CPA composition and stepwise loading protocols remains essential to balance cell viability, functionality, and biosafety. Innovative approaches are further addressing these limitations. Techniques such as nanoparticle-enhanced rewarming and hydrogel encapsulation are enhancing vitrification efficiency, consistently achieving >95% cellular viability while mitigating toxicity risks and maintaining functional competence (Pu et al., 2025; Wang et al., 2016). These advances underscore the ongoing evolution of vitrification protocols toward clinically compliant, highly effective cryopreservation systems.

### 2.5 Chemical strategies to improve cryopreservation

Recent advances in chemical biology have introduced novel strategies to mitigate cryoinjury and enhance cell recovery. First, cell encapsulation within hydrogels or biocompatible polymers has been shown to provide a protective matrix that buffers osmotic fluctuations and minimizes direct contact between cells and ice crystals (Ortiz Silva et al., 2024). Second, intracellular delivery of cryoprotectants has been explored to overcome the limitation of non-permeating CPAs. Approaches such as nanoparticle carriers, membrane transporters, or transient membrane permeabilization can introduce molecules like trehalose or glycerol into the cytosol. This process enhances intracellular protection (Murray et al., 2024; Stewart et al., 2018). Third, caspase inhibitors have demonstrated considerable efficacy in reducing apoptosis triggered by freeze–thaw stress. For example, in umbilical cord–derived MSCs, the broad-spectrum caspase inhibitor Z-VAD-FMK effectively suppressed activation of caspases-3 and -8, inhibited DNA degradation, and reduced cell death post-thaw. Selective inhibitors such as z-IETD-FMK, z-LEHD-FMK, and z-DEVD-FMK specifically attenuate both extrinsic and intrinsic apoptotic pathways, including cleavages such as Bid cleavage, thereby enhancing cell survival (Bissoyi and Pramanik, 2014). Together, these chemical strategies integrate extracellular protection with intracellular signaling modulation, offering a more comprehensive framework to maintain both viability and therapeutic potency of cryopreserved MSCs for clinical applications.

### 2.6 Advanced cryopreservation processes and equipment

Beyond the medium composition, the freezing process itself is a critical variable. Controlled-rate freezers allow for precise, programmed cooling protocols (e.g., 1 °C/min), which are considered the gold standard for optimizing cell survival by carefully managing the balance between dehydration and intracellular ice formation (Duarte Rojas et al., 2024; Abraham and Goel, 2025). Cells that were cryopreserved using programmed freezing consistently showed higher metabolic activity and better-preserved phenotypic characteristics upon thawing compared to simple passive freezing methods (Mohamed et al., 2024).

To meet the demands of clinical translation, the field has moved towards automated, closed-system manufacturing platforms. Systems like the Quantum® Cell Expansion System and the CliniMACS Prodigy® are designed to perform cell culture, expansion, and even formulation in a sterile, GMP-compliant environment, significantly reducing the risk of contamination and human error (Strecanska et al., 2025). In addition to cell processing, controlled-rate freezers and programmable vitrification devices—such as the Kryo 560-16, Planer KRYO 360, and VIA Freeze® systems—are increasingly integrated into these platforms to standardize the cryopreservation process. These automated platforms enhance the efficiency, scalability, and consistency of MSC production, ensuring that large, uniform batches of high-quality cells can be produced for clinical trials and commercial supply (Strecanska et al., 2025). The integration of cryopreservation steps into these automated workflows is a key enabler for the industrialization of cell therapy.

## 3 Effects of cryopreservation on the biological characteristics of MSCs

**FIGURE 4:**
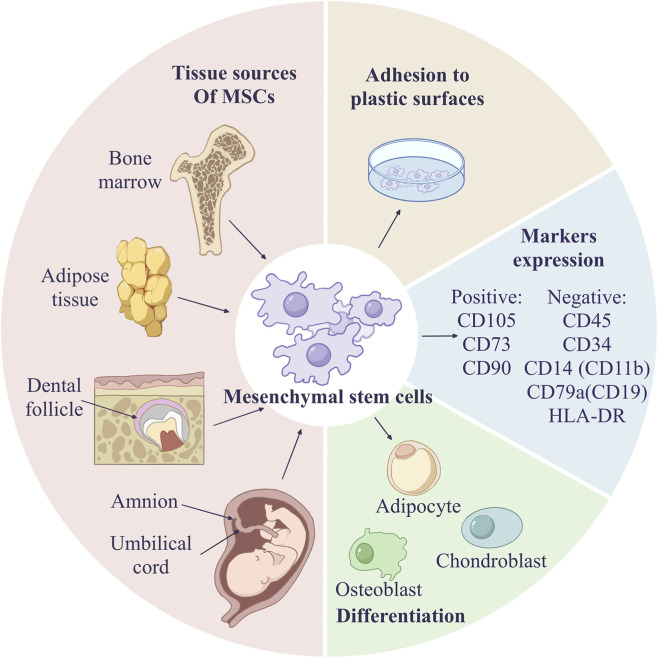
Minimal criteria for defining human MSCs.

Having established the technological foundations of MSC cryopreservation, we now turn to a critical question: how does this process impact the fundamental biological characteristics that define their therapeutic identity? In 2006, the Mesenchymal Stem Cell Committee of the International Society for Cellular Therapy (ISCT) established and published minimal criteria for defining human MSCs: (1) Morphological adherence to plastic culture surfaces with characteristic spindle-shaped or fibroblast-like morphology; (2) Surface antigen expression profile demonstrating ≥95% positivity for CD105, CD73, and CD90, while exhibiting ≤2% positivity for CD45, CD14/CD11b, CD34, CD19/CD79α, and HLA-DR; (3) Multilineage differentiation potential confirmed through *in vitro* induction into osteoblasts, chondrocytes, and adipocytes under defined culture conditions (Dominici et al., 2006) (Figure 4). While modern cryopreservation protocols can achieve high post-thaw viability, it is crucial to understand the more subtle impacts on the biological attributes that define MSCs therapeutic potential (Table 2).

**TABLE 2:** Summary of tissue-origin specific cryopreservation vulnerabilities and their functional implications in MSCs.

MSC tissue source	Key biophysical traits & cryopreservation vulnerabilities	Impact on differentiation potential	Impact on immunomodulatory function	Recommended optimization strategies	Reference
Bone Marrow (BM-MSCs)	• Smaller, spindle-shaped morphology • High sensitivity to DMSO cytotoxicity • Pronounced activation of post-thaw apoptosis pathways	• Osteogenic potential: Moderately to highly sensitive; often shows reduced mineralization • Chondrogenic potential: Relatively well-preserved • Adipogenic potential: Variable reports, generally less affected	• Functionally vulnerable • Significant reduction in IFN-γ-induced IDO activity and PGE2 secretion • Impaired T-cell suppression and regulatory T-cell (Treg) induction	• Use lower DMSO concentrations (e.g., 5%–7.5%) • Supplement with apoptosis inhibitors (e.g., Z-VAD-FMK) • Ensure adequate post-thaw recovery time before functional assessment	Heng, 2009; Bueno et al. (2010)
Adipose Tissue (AD-MSCs)	• Larger cell volume and lower surface-area-to-volume ratio (SA/V) • High susceptibility to intracellular ice formation (IIF) during rapid cooling	• Adipogenic potential: Relatively stable and resilient • Osteogenic potential: Highly sensitive; often significantly impaired • Chondrogenic potential: Moderately affected	• T-cell suppression capacity is partially retained • May exhibit an altered secretome profile, with reductions in key anti-inflammatory factors • Macrophage polarization toward M2 phenotype may be diminished	• Precise control of cooling rate to avoid excessively rapid cooling • Utilize membrane-stabilizing polymers (e.g., PEG) in cryomedium • Explore intracellular delivery of non-penetrating CPAs	Abraham and Goel (2024), Zanata et al. (2016)
Umbilical Cord (UC-MSCs, Wharton’s Jelly)	• High heterogeneity in primary isolates • Robust proliferative capacity, but post-thaw subset composition may shift • Generally show higher post-thaw viability and growth recovery	• Multilineage potential: Typically well-preserved post-thaw, demonstrating strong functional resilience • Often show minimal loss in osteogenic, chondrogenic, and adipogenic capacity compared to other sources	• Often possess a robust innate immunomodulatory capacity • May exhibit higher functional resilience to cryopreservation stress • IDO activity and T-cell suppression can be better maintained with optimized protocols	• Ensure sufficient post-thaw recovery and expansion to restore a stable phenotype • Prioritize functional potency assays (over mere viability) for quality control • Leverage their inherent resilience to develop DMSO-reduced or serum-free formulations	Polchow et al. (2012), Swamynathan et al. (2014)

DMSO: Dimethyl sulfoxide; IDO: Indoleamine 2,3-dioxygenase; PGE2: Prostaglandin E2; SA/V: Surface-area-to-volume ratio; IIF: intracellular ice formation; PEG: polyethylene glycol; CPAs: Cryoprotective agents.

### 3.1 Changes in cell morphology and surface markers

A fundamental requirement is the maintenance of basic cellular characteristics. Most studies report that cryopreserved MSCs, upon thawing and re-culture, retain their characteristic spindle-shaped morphology and exhibit good attachment and proliferation capabilities, similar to their pre-cryopreservation state (Lin et al., 2021). The canonical MSC surface markers (CD73, CD90, CD105) largely maintain their expression levels post-thaw, which serves as a key identity criterion (Wang W. et al., 2022). However, some studies have noted more nuanced changes. For example, one study on rat adipose-derived MSCs (AD-MSCs) found that while the core markers were stable, the expression levels of some functional molecules, such as TGF-β1 and IL-6, were significantly decreased after cryopreservation (Farag et al., 2024). Notably, evidence indicates that cells require a recovery or adaptation phase after thawing, and several studies have shown that without this post-cryopreservation adaptation, the full therapeutic potential of stem cells cannot be restored. Therefore, it is possible that the impairment of functional indicators is alleviated during recovery; however, further investigation is required to confirm this (Linkova et al., 2022). This highlights that stability can vary depending on the specific marker and cell source, with stem cells from dental pulp (hDPSCs) showing high phenotypic stability (Wang W. et al., 2022), while AD-MSCs may exhibit some functional alterations (He et al., 2018).

### 3.2 Impact on multipotent differentiation potential

The hallmark of MSCs is their ability to differentiate into osteoblasts, chondrocytes, and adipocytes. The cryopreservation process can differentially affect these lineage potentials,and this effect is further complicated by the MSC tissue source. Several studies have reported a general decrease in the proliferation and differentiation capacity of cryopreserved MSCs compared to fresh cells, particularly for bone marrow-derived (BM-MSCs) and adipose-derived (AD-MSCs) sources (Pola-Silva et al., 2021; Cottle et al., 2022; Miroslava et al., 2020; Gao et al., 2020). The sensitivity appears to be lineage-specific. For instance, some cryopreservation protocols have been shown to preserve adipogenic potential while significantly reducing osteogenic potential in AD-MSCs (Ntege et al., 2020), whereas BM-MSCs might exhibit a different pattern of vulnerability. Chondrogenic differentiation also appears variably affected, with certain cryopreservation methods causing moderate decreases in cartilage matrix production, indicating that chondrogenic pathways may be partially susceptible to cryo-injury (Ali et al., 2024; Dicks et al., 2023). These observations suggest that cryopreservation can impair specific lineage signaling pathways, making post-thaw functional assessment essential to ensure therapeutic efficacy. The differentiation into more specialized lineages, such as cardiomyocytes, appears to be particularly sensitive. Cardiomyogenic differentiation is a complex process requiring precise signaling cues, which can be disrupted by the stresses of cryopreservation and thawing (Koung et al., 2023; Pilbauerova et al., 2022). The underlying mechanisms may involve cryo-induced dysfunction in key signaling pathways (e.g., Wnt, TGF-β), increased intracellular oxidative stress, or a decline in the cells’ overall self-renewal capacity, all of which can compromise their ability to respond to differentiation stimuli (Cottle et al., 2022; Kanazawa et al., 2022; El Assaad et al., 2024). This is a critical consideration for cardiac regeneration therapies, where ensuring the preservation of this specific potential is paramount.

### 3.3 Impact on immunomodulatory capacity

The paracrine-mediated immunomodulatory function is arguably the most important therapeutic mechanism of MSCs. Cryopreservation can impact this function by altering the secretome and the cells’ responsiveness to inflammatory signals (Table 3).

**TABLE 3:** Comparative analysis of the immunomodulatory function of fresh versus cryopreserved MSCs.

Cellular function/Molecule	Observation in fresh MSCs	Observed impact of cryopreservation	Proposed mechanisms for cryopreservation-induced changes	Reference
Indoleamine 2,3-dioxygenase (IDO)	Robust expression and activity upon IFN-γ licensing	Inconsistent (↓➚↔); Often shows reduced activity; Some studies report transient stress-induced upregulation	Impaired response to inflammatory licensing (IFN-γ signaling); General disruption of protein synthesis and metabolic pathways post-thaw	Moll et al. (2016)
Prostaglandin E2 (PGE2)	High constitutive and induced synthesis	Consistently decreased (↓) secretion	Cryo-damage to continuous enzymatic synthesis pathways (COX-2); Mitochondrial dysfunction affecting energy-dependent processes	Chinnadurai et al. (2016)
Transforming Growth Factor-β (TGF-β)	Constitutive secretion, crucial for Treg induction	Frequently reduced (↓)	Alterations in the cytoskeleton and secretory machinery; Potential depletion during the freeze-thaw stress response	de Witte et al. (2017)
Human Leukocyte Antigen-G (HLA-G)	Surface and soluble expression, induces immune tolerance	Can be compromised (↓)	Sensitivity of membrane-bound molecules to ice crystal and osmotic damage; Shedding or altered trafficking	Rui et al. (2016)
Overall T-cell Proliferation Suppression	Potent suppression in co-culture assays (e.g., MLR)	Generally attenuated but retained (↓); A quantifiable reduction in suppressive potency is common	Cumulative result of reduced secretion of key soluble factors (IDO, PGE2, TGF-β); Altered cell surface co-stimulatory molecule expression	Wang et al. (2014)
Regulatory T-cell (Treg) Induction	Efficiently promotes the expansion of CD4^+^CD25+FOXP3+ Tregs	Frequently impaired (↓)	Primarily linked to the reduced production of Treg-polarizing factors like TGF-β and PGE2	Galipeau (2013)
T-helper 1 (Th1) & 17 (Th17) Suppression	Effectively inhibits pro-inflammatory IFN-γ (Th1) and IL-17 (Th17) production	Suppression efficiency is often reduced (↓)	Weakened paracrine signaling fails to fully counteract Th1/Th17 polarization signals in the microenvironment	Luz-Cra et al. (2013)
Macrophage Polarization	Promotes a shift from pro-inflammatory M1 to anti-inflammatory M2 phenotype	Capability is often diminished (↓)	Reduced secretion of key mediators like PGE2 and other soluble factors that drive M2 polarization	Swamynathan et al. (2014)

The impact of cryopreservation on the secretory profile of MSCs is both complex and pivotal. MSC expression of indoleamine 2,3-dioxygenase (IDO), a key molecule in suppressing inflammation, appears to vary with cryopreservation method. Some studies report an initial increase in IDO expression and activity, which might be an immediate stress response. This upregulation may decrease over longer culture periods (Tan et al., 2019). In contrast, other investigations demonstrate a significant reduction in IDO-mediated T-cell suppression, highlighting protocol-dependent variability (François et al., 2012). The synthesis of prostaglandin E2 (PGE2), another critical mediator, is highly dependent on continuous enzymatic activity. Cryoinjury can disrupt this biosynthetic pathway, frequently leading to a marked decrease in PGE2 secretion (Shi et al., 2018). Similarly, the production of key immunoregulatory molecules, such as transforming growth factor-beta (TGF-β) and human leukocyte antigen-G (HLA-G), can be compromised (Hoogduijn et al., 2016).

Beyond changes in TGF-β1 and IL-6, recent studies have directly assessed the effects of cryopreservation on MSCs’ immunomodulatory capacity. Functional assays measuring the suppression of T-cell proliferation, considered a gold-standard assessment, consistently indicate that cryopreserved MSCs generally retain their immunosuppressive capacity, albeit often with a quantifiable reduction in potency compared to their fresh counterparts (Vymetalova et al., 2020), showing impaired suppression of T-cell proliferation, reduced induction of regulatory T cells, and altered cytokine-mediated crosstalk with immune cells (François et al., 2012; Pollock et al., 2015). A primary immunomodulatory mechanism of MSCs is the induction of regulatory T-cells (Tregs). While thawed MSCs can still promote Treg expansion, this capability is frequently attenuated (Bárcia et al., 2017). Furthermore, the capacity of cryopreserved MSCs to modulate the balance between pro-inflammatory and anti-inflammatory T-helper cells is preserved but suboptimal. They typically maintain the ability to suppress the polarization of pro-inflammatory Th1 and Th17 cells; however, the efficiency of this suppression may be less pronounced than in fresh MSCs (Le Nail et al., 2018). Cryopreservation may compromise the immunomodulatory function of MSCs, arguably their most important therapeutic role. This is reflected by reduced production of key signaling molecules such as TGF-β1 and IL-6 after thawing (Farag et al., 2024), which could weaken their overall immunosuppressive effect (Farag et al., 2024). For example, diminished post-thaw MSCs activity has been linked to reduced inhibition of effector T-cell responses and altered modulation of macrophage polarization, highlighting a direct compromise of their immunoregulatory potential (Duffy et al., 2011; Li et al., 2023). Studies show that a reduction in TGF-β1 might impair their ability to control T-cell responses, while changes in IL-6 could disrupt their balancing act between pro- and anti-inflammatory signals (Martín-López et al., 2023; Gil-Chinchilla et al., 2024). On the other hand, some work has shown that engineering MSCs to produce more IL-6 actually boosts their therapeutic effect (Huang et al., 2022). This suggests that finding ways to maintain or even enhance key factor expression post-thaw is a promising path forward. Protecting this specific function during the freeze-thaw cycle remains a major research priority.

## 4 How cryopreserved MSCs perform in vivo

While *in vitro* assays are essential, the ultimate test of cryopreserved MSCs occurs in living organisms. The following section synthesizes their performance across diverse preclinical disease models and clinical trials, with a particular focus on the comparison between fresh and cryopreserved cells.

A pivotal consideration, often highlighted by comparative studies, is the functional differences between fresh and cryopreserved MSCs. Interspecies differences (e.g., between rodent and human MSCs) are important. However, directly comparing fresh and cryopreserved aliquots from the same donor source more accurately reveals the true impact of the freeze-thaw process on therapeutic potential. In terms of efficacy, numerous reports indicate that cryopreserved MSCs may exhibit impaired *in vivo* performance compared to their fresh counterparts, including reduced engraftment efficiency, shorter persistence at injury sites, and attenuated therapeutic effects in pre-clinical models of inflammatory diseases (Tan et al., 2019; Le Nail et al., 2018). This likely results from the combined effects of cryoinjury on cell viability, homing receptor integrity, and paracrine factor secretion, as discussed in Section 3.3. Regarding safety, the cryopreservation process itself introduces additional variables. The necessity for penetrating CPAs, most notably DMSO, raises concerns about infusion-related toxicity and potential adverse effects on patients, which are not a factor for freshly transplanted cells (Windrum et al., 2005). Furthermore, the risk of phenotypic drift or the selection of a specific subpopulation during freeze-thaw could, in theory, alter the long-term safety profile, though evidence for this is still emerging. Most critically, the issue of potency—a quantitative measure of a product’s biological activity—is central. Regulatory guidelines emphasize the need for potency assays, yet defining a universal assay for thawed MSCs remains challenging. A cryopreserved batch may meet release criteria based on viability but may possess significantly reduced immunosuppressive or pro-regenerative capacity compared to the pre-freeze culture.

### 4.1 Cardiovascular and respiratory diseases

In the context of cardiac repair using MSCs, clinical results have shown mixed outcomes, reflecting variable efficacy across studies. A randomized controlled trial enrolled 30 patients with chronic ischemic cardiomyopathy who received transendocardial injections of either autologous or allogeneic cryopreserved bone marrow-derived MSCs and reported their outcomes at 6-month follow-up. At 6-month follow-up, left ventricular ejection fraction (LVEF) increased by 4.9% (autologous) and 5.4% (allogeneic), with concomitant significant reduction in myocardial infarct size. No clinically significant alloimmune reactions were observed in either cohort (Ramireddy et al., 2017). A meta-analysis of clinical trials found that cryopreserved umbilical cord MSCs could produce a significant, though often short-lived, improvement in LVEF, especially if post-thaw viability was high (Safwan et al., 2025). The main challenge lies in sustaining the improvement in cardiac function over the long term. At a mechanistic level, these cells have been shown to encourage the expression of cardiac proteins like Troponin T, helping to improve heart muscle function (Clavellina et al., 2023; Ali et al., 2025). However, these outcomes cannot be unequivocally attributed to the act of cryopreservation or the resultant post-thaw cell state, as variability in therapeutic efficacy may also be influenced by factors such as MSCs source, patient selection criteria, and clinical protocol design. More conclusive evidence regarding the impact of cryopreservation on therapeutic efficacy would require direct comparative studies between fresh and cryopreserved MSCs, which would better clarify whether reduced long-term benefit arises specifically from cryoinjury or from other confounding variables (Fan et al., 2020).

### 4.2 Respiratory diseases

Cryopreserved MSCs retain significant therapeutic potential in pulmonary injury models, including pulmonary fibrosis and acute lung injury. Researchers established a cryobank containing 124 cryopreserved aliquots of lung-derived MSCs (L-MSCs) from *Ovis aries* fetuses. *In vitro* differentiation assays confirmed L-MSCs’ trilineage mesodermal differentiation capacity. Crucially, post-thaw L-MSCs maintained anti-fibrotic functionality after undergoing hepatic differentiation. This was evidenced by sustained secretion of urea and albumin, indicating their regenerative potential for pulmonary tissue repair and fibrotic mitigation (Dominguez-Pinilla et al., 2025). The cells have shown clearer promise in acute lung injury models like Ventilator-induced lung injury (VILI). Cryopreserved umbilical cord-derived mesenchymal stem cells (UC-MSCs) exert therapeutic effects through paracrine secretion of multifactorial cytokines and bioactive mediators, effectively mitigating inflammatory responses and facilitating pulmonary tissue regeneration (Dominguez-Pinilla et al., 2025; Mallis et al., 2022). After thawing, they still produce key immunomodulatory factors like IL-6 and IL-10 and can steer the lung’s immune environment toward an anti-inflammatory M2 macrophage phenotype (Mallis et al., 2022; Budgude et al., 2021). This confirms that their essential anti-inflammatory and paracrine functions were well preserved following cryopreservation, supporting their use in treating acute pulmonary conditions.

### 4.3 Kidney diseases

For chronic conditions like diabetic nephropathy (DN), cryopreserved MSCs are used to fight ongoing inflammation and fibrosis. In mouse models of DN, MSCs treatment lowered inflammatory cytokine levels in the kidneys and reduced scarring (Zhu et al., 2025; Wu et al., 2025). This effect is tied to their ability to home to the damaged kidney and shift the local immune cells toward an anti-inflammatory state (Chen et al., 2025; Bejugam et al., 2025). Researchers are also exploring ways to boost their efficacy, for example, by pre-treating the cells with GLP-1 receptor agonists before freezing, which enhances their therapeutic effects in DN models (Liu et al., 2024; Habib et al., 2021).

### 4.4 Graft-versus-host disease

Graft-versus-host disease (GVHD) is a serious complication following allogeneic hematopoietic stem cell transplantation, triggered by donor immune cells attacking recipient tissues. Patients with steroid-refractory acute GVHD (SR-aGVHD) have a particularly poor prognosis (Moreno and Cid, 2019). In 2020, the U.S. FDA approved the first mesenchymal stromal cell (MSC)-based therapy, Ryoncil® (remestemcel-L), for the treatment of pediatric SR-aGVHD, representing a major breakthrough in cellular therapy for this condition (ICHGCP, 2025). Critically, this “off-the-shelf” therapy is administered via intravenous infusion of MSCs immediately after thawing from cryopreserved stocks, underscoring the pivotal role of an effective cryopreservation protocol in enabling its practical clinical application. This therapy acts through multi-dimensional immunomodulatory mechanisms, including the secretion of anti-inflammatory factors, suppression of excessive T-cell activation, and promotion of regulatory T-cell generation, thereby reestablishing immune tolerance. Pivotal Phase III clinical trials demonstrated an overall response rate of 69% among treated patients, with a complete response rate of 47%, significantly improving survival rates. This approval not only provides the first “off-the-shelf” cellular therapeutic option for SR-aGVHD and advances the industrialization of cell therapies, but also lays the foundation for broader clinical applications of MSCs in other immune-related diseases (Kurtzberg et al., 2020). However, challenges such as heterogeneity in treatment response, standardization of manufacturing, and long-term safety require further investigation.

### 4.5 Other diseases

The applications extend beyond these common diseases. In veterinary medicine, they are used to treat systemic inflammation in horses (Uberti et al., 2022; MacDonald and Barrett, 2019). A particularly striking example comes from dentistry, where cryopreserved allogeneic bone marrow MSCs were used to regenerate pulp-like tissue in the necrotic teeth of children, restoring sensitivity and healing lesions (Gomez-Sosa et al., 2024). This case highlights the robust regenerative power these cells can retain even after cryopreservation.

## 5 From lab to clinic: manufacturing and quality control

The translation of cryopreserved MSCs from a laboratory reagent to a reliable clinical product necessitates rigorous standardization and quality control. This final section addresses the manufacturing, regulatory, and logistical frameworks that underpin their successful clinical application.

### 5.1 GMP regulations and oversight

GMP regulations are non-negotiable; they are designed to ensure every batch of a cell therapy product is safe, pure, potent, and consistent (Bio-Techne, 2025). This means having tight control over the entire process, from screening donors to the final storage of the cryopreserved cells (Li et al., 2024). Key quality metrics for a cryopreserved MSC product are cell viability, purity, identity, and functional potency. Although a post-thaw viability above 70%–80% is a common release criterion, it alone does not guarantee product quality (Rusconi et al., 2024). Purity tests must confirm the absence of unwanted cells, and identity tests ensure the product actually contains MSCs. Importantly, studies have shown that MSCs produced under these strict GMP conditions can be just as functional as their research-grade counterparts, proving that high-quality cells can be made at scale (Mendt et al., 2021).

### 5.2 Quality assessment of cryopreserved MSCs in preclinical and clinical trials

A central challenge in the field is defining and measuring attributes that correlate with clinical efficacy. As discussed, post-thaw viability is a necessary but insufficient metric (Putra et al., 2021). This is underpinned by the complex relationship between pre-freeze and post-thaw cell status. A high pre-freeze viability (>90%) is a prerequisite for a successful outcome; however, the freeze-thaw process itself can induce a significant viability drop of 10%–30% due to inherent cryoinjury, meaning that even an optimal starting point does not guarantee a high-quality final product (El Kadi et al., 2025). The current focus is on potency assays that measure the product’s mechanism of action for specific indications, such as immunomodulatory capacity or pro-angiogenic activity (Dave et al., 2022). A systematic review found that while there were often no significant differences in vitro potency between cryopreserved and fresh MSCs, the *in vivo* efficacy could vary, highlighting a gap in our understanding (Dave et al., 2022). Furthermore, batch-to-batch consistency is paramount. Variations in cell source, culture conditions, and cryopreservation protocols can impact cell function and therapeutic outcomes (Wiese et al., 2022). For example, in cardiovascular trials, MSCs efficacy has been shown to correlate with patient-specific factors like baseline LVEF and inflammatory status, suggesting that a successful therapy will require both a consistent product and patient stratification (Patel et al., 2025). Establishing a clear correlation between *in vitro* quality attributes and clinical outcomes remains a key goal in the field.

### 5.3 Cryopreserved cell preparations: storage, transportation, thawing and recovery, and handling

Ultra-low temperature cryopreservation is used for storing cryopreserved preparations. Liquid nitrogen (−196 °C) or mechanical refrigeration systems (−150 °C to −80 °C) maintain cell viability for extended periods (Harris, 2014). The logistics involved in transporting frozen products, commonly referred to as the “vial-to-vein” process, is of crucial importance. This necessitates a robust cold chain management system. Prior to transportation, stem cells from different sources require customized formulations of cryoprotectants to prevent ice crystal damage caused by temperature fluctuations during transportation (Harris, 2014). During transportation, the products must remain at ultra-low temperatures (for example, in dry ice or liquid nitrogen vapor shippers) until they reach the clinical site (Weng, 2023; Wang et al., 2023; Heydarzadeh et al., 2022). Furthermore, real-time temperature monitoring and the utilization of shock-absorbing packaging can be employed (Harris, 2014).

At the bedside, a standardized operating procedure (SOP) for thawing is crucial. Moving beyond the conventional 37 °C water bath, which poses contamination risks and variability, standardized and qualified thawing systems are now considered best practice. Automated, closed-system dry-thaw devices are increasingly adopted in GMP settings. These devices precisely control the warming rate at ∼100 °C/min, ensuring a consistent and rapid thaw that maximizes cell recovery and minimizes the toxic effects of DMSO(15). This rapid thawing minimizes ice recrystallization damage (Weng, 2023). Post-thaw, the cell product must be administered within a short, validated timeframe to ensure viability and function are not compromised (Harris, 2014). Moreover, precise alignment between thawing operations and clinical treatment plans should be achieved, and the entire management process from storage to clinical application must be subject to strict control and documentation, so as to ensure product quality and patient safety (Harris, 2014; Kumar et al., 2024).

Finally, the latest frontier in ‘vial-to-vein’ logistics focuses on ensuring product quality at the moment of infusion. While post-thaw viability via trypan blue remains common, it is a poor predictor of function. Emerging technologies are enabling more sophisticated, rapid assessments at the point-of-care. Portable flow cytometers can now be deployed to provide a rapid immunophenotype confirmation within minutes of thawing (Gao et al., 2021). Furthermore, in-line viability sensors based on dielectric spectroscopy are under development for real-time, label-free monitoring of cell concentration and vitality during the thaw-and-wash process (Rosell-Valle et al., 2021).

## 6 Discussion and future perspectives

The significance of cryopreservation technology in the clinical translation of MSCs is increasingly prominent. Current research indicates that cryopreserved MSCs maintain basic biological characteristics and multi-directional differentiation potential; however, they still exhibit some functional decline, particularly in immunomodulation (Medrano-Trochez et al., 2021; Kamprom et al., 2024). Although clinical trials have demonstrated the safety and therapeutic potential of cryopreserved MSCs, inconsistent results and limited data have hindered their widespread application.

To develop MSCs as standardized, “off-the-shelf” therapeutics, researchers must overcome two main challenges: preserving post-thaw functionality and managing biological heterogeneity. The advancements synthesized in this review illuminate a clear, integrated path forward. A primary obstacle, the cytotoxicity of DMSO, is being addressed by two compelling strategies. Importantly, it should be noted that low concentrations of DMSO (≤5–10%) are generally considered clinically acceptable when carefully removed prior to administration, thereby mitigating acute toxicity risks (Santos et al., 2024). In parallel, another translational barrier arises from the xenogeneic components of FBS, which may introduce risks of immunogenicity and pathogen transmission (Martin et al., 2022; Dessels et al., 2016). To address this, serum-free and chemically defined cryomedia have been developed, while HPL has emerged as a clinically safer alternative to FBS, providing comparable growth factor support without xenogeneic concerns. These approaches collectively strengthen the clinical feasibility of MSCs cryopreservation by reducing both solvent-related cytotoxicity and serum-derived safety risks.

Three main approaches address these challenges: 1) rational design of synthetic, biocompatible CPAs, such as trehalose-based polyethers with IRI activity (El Kadi et al., 2025) and zwitterionic polymers that manage osmotic stress (Dave et al., 2022); 2) preserving MSCs within their native tissue “niche,” leveraging the natural extracellular matrix as a superior cryoprotective scaffold (Yang et al., 2016); and 3) upgrading equipment and technologies to enhance functional recovery by optimizing vitrification methods and developing advanced cryopreservation processes and devices. These innovations are paving the way for safer, more effective cryopreservation protocols (Strecanska et al., 2025; Wang et al., 2011).

To overcome the limitations of traditional, non-specific identity markers, the field is adopting robust core transcriptomic signatures. These signatures provide a definitive molecular fingerprint for MSCs and remain stable across manufacturing pressures (Wiese and Braid, 2020). Beyond identity, assessing therapeutic potency is shifting from simple viability counts to a matrix of validated, indication-specific functional assays. Rigorously validated methods like the mixed lymphocyte reaction (MLR) for quantifying immunomodulation (Nicotra et al., 2020) and advanced microphysiological systems (MPS) for measuring vasculogenic capacity exemplify this crucial trend (Lam et al., 2022). These assays provide the necessary tools to ensure batch-to-batch consistency and characterize products for their intended clinical use.

In synthesis, the path towards effective, off-the-shelf MSCs therapies requires an integrated quality control framework that incorporates: 1) advanced cryopreservation techniques using novel CPAs or niche-preserving storage; 2) robust molecular signatures for unambiguous cell identity; and 3) a panel of validated, indication-specific potency assays. However, despite clear progress, significant challenges remain. The foremost challenge is establishing a definitive correlation between *in vitro* potency measurements and *in vivo* clinical efficacy. This correlation remains the “holy grail” for the field. To bridge this gap, large-scale clinical trials linking detailed product characterization to patient outcomes are urgently needed. Furthermore, standardizing these advanced assays across different laboratories is essential. The development of cost-effective, rapid surrogate markers is also critical for practical implementation in a GMP manufacturing setting. Ultimately, the successful integration of these innovations in cryobiology and bio-analytics will be the key to delivering MSCs therapies with the predictable, consistent, and potent therapeutic benefits that patients require.

## Glossary

MSCs: mesenchymal stem cells
CPAs: cryoprotective agents
GVHD: Graft-versus-Host Disease
QC: quality control
IRI: ice recrystallization inhibition
ECM: extracellular matrix
DMSO: dimethyl sulfoxide
FBS: fetal bovine serum
HPL: human platelet lysate
PEG: polyethylene glycol
BSA: bovine serum albumin
SCF: stem cell factor
EG: ethylene glycol
PVA: polyvinyl alcohol
UCB-MSCs: umbilical cord blood-derived MSCs
ISCT: International Society for Cellular Therapy
AD-MSCs: adipose-derived MSCs
hDPSCs: human dental stem cells
BM-MSCs: bone marrow-derived MSCs
LVEF: left ventricular ejection fraction
L-MSCs: lung-derived MSCs
UC-MSCs: umbilical cord-derived mesenchymal stem cells
DN: diabetic nephropathy
SOP: standardized operating procedure
MLR: mixed lymphocyte reaction
MPS: microphysiological systems
IDO: Indoleamine 2,3-dioxygenase
PGE2: Prostaglandin E2
SA/V: Surface-area-to-volume ratio
IIF: Intracellular ice formation
VILI: Ventilator-induced lung injury

## References

[B1] Abraham M. Goel S. (2024). Comprehensive characterisation and cryopreservation optimisation of buffalo (Bubalus bubalis) adipose tissue-derived mesenchymal stem cells. Cryobiology 115, 104896. 10.1016/j.cryobiol.2024.104896 38641158

[B2] Abraham M. Goel S. (2025). Species-specific optimisation of cryopreservation media for goat and buffalo adipose-derived mesenchymal stem cells. Cryobiology 118, 105211. 10.1016/j.cryobiol.2025.105211 39921190

[B3] AlHindi M. Philip M. R. (2021). Osteogenic differentiation potential and quantification of fresh and cryopreserved dental follicular stem cells-an *in vitro* analysis. J. Stem Cells Regen. Med. 17 (1), 28–34. 10.46582/jsrm.1701004 34434005 PMC8372412

[B4] Ali E. A. M. Smaida R. Meyer M. Ou W. Li Z. Han Z. (2024). iPSCs chondrogenic differentiation for personalized regenerative medicine: a literature review. Stem Cell Res. Ther. 15 (1), 185. 10.1186/s13287-024-03794-1 38926793 PMC11210138

[B5] Ali S. A. Mahmood Z. Mubarak Z. Asad M. Sarfraz Chaudhri M. T. Bilal L. (2025). Assessing the potential benefits of stem cell therapy in cardiac regeneration for patients with ischemic heart disease. Cureus 17 (1), e76770. 10.7759/cureus.76770 39897258 PMC11786102

[B6] Andreoli V. Berni P. Conti V. Ramoni R. Basini G. Grolli S. (2024). Mesenchymal stromal cells derived from canine adipose tissue: evaluation of the effect of different shipping vehicles used for clinical administration. Int. J. Mol. Sci. 25 (6), 3426. 10.3390/ijms25063426 38542399 PMC10970639

[B7] Bárcia R. N. Santos J. M. Teixeira M. Filipe M. Pereira A. R. S. Ministro A. (2017). Umbilical cord tissue-derived mesenchymal stromal cells maintain immunomodulatory and angiogenic potencies after cryopreservation and subsequent thawing. Cytotherapy 19 (3), 360–370. 10.1016/j.jcyt.2016.11.008 28040463

[B8] Bejugam D. Bu S. Nguyen A. N. Yaltaghian M. Smolen K. K. (2025). New frontiers in type I diabetes treatment: the impact of mesenchymal stromal cells on long-term complications. Front. Clin. Diabetes Healthc. 6, 1586061. 10.3389/fcdhc.2025.1586061 40458682 PMC12127150

[B9] Bio-Techne (2025). GMP raw materials: meeting the demand for cell therapy. Bio-Techne. Available online at: https://www.bio-techne.com/resources/blogs/gmp-raw-materials-meeting-the-cell-therapy-demand. (Accessed September 7, 2025).

[B10] Bissoyi A. Pramanik K. (2014). Role of the apoptosis pathway in cryopreservation-induced cell death in mesenchymal stem cells derived from umbilical cord blood. Biopreservation Biobanking 12 (4), 246–254. 10.1089/bio.2014.0005 25162461

[B11] Bojic S. Murray A. Bentley B. L. Spindler R. Pawlik P. Cordeiro J. L. (2021). Winter is coming: the future of cryopreservation. BMC Biol. 19 (1), 56. 10.1186/s12915-021-00976-8 33761937 PMC7989039

[B12] Budgude P. Kale V. Vaidya A. (2021). Cryopreservation of mesenchymal stromal cell-derived extracellular vesicles using trehalose maintains their ability to expand hematopoietic stem cells *in vitro* . Cryobiology 98, 152–163. 10.1016/j.cryobiol.2020.11.009 33253747

[B13] Bueno C. Montes R. Menendez P. (2010). The ROCK inhibitor Y-27632 negatively affects the expansion/survival of both fresh and cryopreserved cord blood-derived CD34+ hematopoietic progenitor cells: Y-27632 negatively affects the expansion/survival of CD34+HSPCs. Stem Cell Rev. Rep. 6 (2), 215–223. 10.1007/s12015-010-9118-5 20180051

[B14] Capicciotti C. J. Kurach J. D. R. Turner T. R. Mancini R. S. Acker J. P. Ben R. N. (2015). Small molecule ice recrystallization inhibitors enable freezing of human red blood cells with reduced glycerol concentrations. Sci. Rep. 5, 9692. 10.1038/srep09692 25851700 PMC4389209

[B15] Caplan A. I. (2017). Mesenchymal stem cells: time to change the name. Stem Cells Transl. Med. 6 (6), 1445–1451. 10.1002/sctm.17-0051 28452204 PMC5689741

[B16] Chang T. Zhao G. (2021). Ice inhibition for cryopreservation: materials, strategies, and challenges. Adv. Sci. Weinh Baden-Wurtt Ger. 8 (6), 2002425. 10.1002/advs.202002425 33747720 PMC7967093

[B17] Chen C. Xu B. Li W. Chen J. Yang M. Gao L. (2025). New perspectives on the treatment of diabetic nephropathy: challenges and prospects of mesenchymal stem cell therapy. Eur. J. Pharmacol. 998, 177543. 10.1016/j.ejphar.2025.177543 40139419

[B18] Chinnadurai R. Copland I. B. Garcia M. A. Petersen C. T. Lewis C. N. Waller E. K. (2016). Cryopreserved mesenchymal stromal cells are susceptible to T-Cell mediated apoptosis which is partly rescued by IFNγ licensing. Stem Cells Dayt Ohio. 34 (9), 2429–2442. 10.1002/stem.2415 27299362 PMC5016228

[B19] Cimino M. Gonçalves R. M. Barrias C. C. Martins M. C. L. (2017). Xeno-free strategies for safe human mesenchymal stem/Stromal cell expansion: supplements and coatings. Stem Cells Int. 2017, 6597815. 10.1155/2017/6597815 29158740 PMC5660800

[B20] Clavellina D. Balkan W. Hare J. M. (2023). Stem cell therapy for acute myocardial infarction: Mesenchymal stem Cells and induced pluripotent stem cells. Expert Opin. Biol. Ther. 23 (10), 951–967. 10.1080/14712598.2023.2245329 37542462 PMC10837765

[B21] Cottle C. Porter A. P. Lipat A. Turner-Lyles C. Nguyen J. Moll G. (2022). Impact of cryopreservation and freeze-thawing on therapeutic properties of mesenchymal stromal/stem cells and other common cellular therapeutics. Curr. Stem Cell Rep. 8 (2), 72–92. 10.1007/s40778-022-00212-1 35502223 PMC9045030

[B22] Crow D. (2019). Could iPSCs enable “Off-the-Shelf” cell therapy? Cell 177 (7), 1667–1669. 10.1016/j.cell.2019.05.043 31199910

[B23] Dave C. Mei S. H. J. McRae A. Hum C. Sullivan K. J. Champagne J. (2022). Comparison of freshly cultured *versus* cryopreserved mesenchymal stem cells in animal models of inflammation: a pre-clinical systematic review. eLife 11, e75053. 10.7554/eLife.75053 35838024 PMC9286731

[B24] de Camargo G. C. da Cruz Landim-Alvarenga F. Maciel A. P. Dos Santos D. B. de Paula Freitas Dell’Aqua C. E Alvarenga M. L. (2025). Polyvinyl alcohol can replace the fetal bovine serum during cryopreservation of canine adipose mesenchymal stromal cells. Vitro Cell Dev. Biol. Anim. 61 (4), 369–373. 10.1007/s11626-025-01046-x 40399738

[B25] de Witte S. F. H. Lambert E. E. Merino A. Strini T. Douben HJCW O’Flynn L. (2017). Aging of bone marrow- and umbilical cord-derived mesenchymal stromal cells during expansion. Cytotherapy 19 (7), 798–807. 10.1016/j.jcyt.2017.03.071 28462821

[B26] Dessels C. Potgieter M. Pepper M. S. (2016). Making the switch: alternatives to fetal bovine serum for adipose-derived stromal cell expansion. Front. Cell Dev. Biol. 4, 115. 10.3389/fcell.2016.00115 27800478 PMC5065960

[B27] Dias C. Commin L. Bonnefont-Rebeix C. Buff S. Bruyère P. Trombotto S. (2023). Comparative evaluation of the *in vitro* cytotoxicity of a series of chitosans and chitooligosaccharides water-soluble at physiological pH. Polymers 15 (18), 3679. 10.3390/polym15183679 37765533 PMC10537996

[B28] Dicks A. R. Steward N. Guilak F. Wu C. L. (2023). Chondrogenic differentiation of human-induced pluripotent stem cells. Methods Mol. Biol. Clifton NJ. 2598, 87–114. 10.1007/978-1-0716-2839-3_8 36355287 PMC9830630

[B29] Ding D. C. Shyu W. C. Lin S. Z. (2011). Mesenchymal stem cells. Cell Transpl. 20 (1), 5–14. 10.3727/096368910X 21396235

[B30] Dominguez-Pinilla N. González-Granado L. I. Gonzaga A. López Diaz M. Castellano Yáñez C. Aymerich C. (2025). Consecutive intrabronchial administration of Wharton’s jelly-derived mesenchymal stromal cells in ECMO-supported pediatric patients with end-stage interstitial lung disease: a safety and feasibility study (CIBA method). Stem Cell Res. Ther. 16 (1), 164. 10.1186/s13287-025-04289-3 40188166 PMC11972491

[B31] Dominici M. Le Blanc K. Mueller I. Slaper-Cortenbach I. Marini F. Krause D. (2006). Minimal criteria for defining multipotent mesenchymal stromal cells. The international society for cellular therapy position statement. Cytotherapy 8 (4), 315–317. 10.1080/14653240600855905 16923606

[B32] Duarte Rojas J. M. Restrepo Múnera L. M. Estrada M. S. (2024). Comparison between platelet lysate, platelet lysate serum, and fetal bovine serum as supplements for cell culture, expansion, and cryopreservation. Biomedicines 12 (1), 140. 10.3390/biomedicines12010140 38255245 PMC10813006

[B33] Duffy M. M. Ritter T. Ceredig R. Griffin M. D. (2011). Mesenchymal stem cell effects on T-cell effector pathways. Stem Cell Res. Ther. 2 (4), 34. 10.1186/scrt75 21861858 PMC3219065

[B34] El Assaad N. Chebly A. Salame R. Achkar R. Bou Atme N. Akouch K. (2024). Anti-aging based on stem cell therapy: a scoping review. World J. Exp. Med. 14 (3), 97233. 10.5493/wjem.v14.i3.97233 39312703 PMC11372738

[B35] El Kadi K. Murad S. Janajreh I. (2025). Ice crystallization kinetics in supercooled droplets from a molecular perspective. J. Colloid Interface Sci. 703 (Pt 2), 139192. 10.1016/j.jcis.2025.139192 41100921

[B36] Fahy G. M. (1986). Vitrification: a new approach to organ cryopreservation. Prog. Clin. Biol. Res. 224, 305–335. 3540994

[B37] Fan X. L. Zhang Y. Li X. Fu Q. L. (2020). Mechanisms underlying the protective effects of mesenchymal stem cell-based therapy. Cell Mol. Life Sci. CMLS 77 (14), 2771–2794. 10.1007/s00018-020-03454-6 31965214 PMC7223321

[B38] Farag A. Ngeun S. K. Kaneda M. Aboubakr M. Elhaieg A. Hendawy H. (2024). Exploring the potential effects of cryopreservation on the biological characteristics and cardiomyogenic differentiation of rat adipose-derived mesenchymal stem cells. Int. J. Mol. Sci. 25 (18), 9908. 10.3390/ijms25189908 39337396 PMC11432599

[B39] François M. Copland I. B. Yuan S. Romieu-Mourez R. Waller E. K. Galipeau J. (2012). Cryopreserved mesenchymal stromal cells display impaired immunosuppressive properties as a result of heat-shock response and impaired interferon-γ licensing. Cytotherapy 14 (2), 147–152. 10.3109/14653249.2011.623691 22029655 PMC3279133

[B40] Freimark D. Sehl C. Weber C. Hudel K. Czermak P. Hofmann N. (2011). Systematic parameter optimization of a Me(2)SO- and serum-free cryopreservation protocol for human mesenchymal stem cells. Cryobiology 63 (2), 67–75. 10.1016/j.cryobiol.2011.05.002 21620818

[B41] Galipeau J. (2013). The mesenchymal stromal cells dilemma--does a negative phase III trial of random donor mesenchymal stromal cells in steroid-resistant graft-versus-host disease represent a death knell or a bump in the road? Cytotherapy 15 (1), 2–8. 10.1016/j.jcyt.2012.10.002 23260081

[B42] Gao S. Ogawa M. Takami A. Takeshita K. Kato H. (2020). Practical and safe method of long-term cryopreservation for clinical application of human adipose-derived mesenchymal stem cells without a programmable freezer or serum. Cryo Lett. 41 (6), 337–343. 33990810

[B43] Gao L. Zhou Q. Zhang Y. Sun S. Lv L. Ma P. (2021). Dimethyl sulfoxide-free cryopreservation of human umbilical cord mesenchymal stem cells based on zwitterionic betaine and electroporation. Int. J. Mol. Sci. 22 (14), 7445. 10.3390/ijms22147445 34299064 PMC8306716

[B44] Gil-Chinchilla J. I. Bueno C. Martínez C. M. Ferrández-Múrtula A. García-Hernández A. M. Blanquer M. (2024). Optimizing cryopreservation conditions for use of fucosylated human mesenchymal stromal cells in anti-inflammatory/immunomodulatory therapeutics. Front. Immunol. 15, 1385691. 10.3389/fimmu.2024.1385691 38605955 PMC11007032

[B45] Gomez-Sosa J. F. Cardier J. E. Wittig O. Díaz-Solano D. Lara E. Duque K. (2024). Allogeneic bone marrow mesenchymal stromal cell transplantation induces dentin pulp complex-like formation in immature teeth with pulp necrosis and apical periodontitis. J. Endod. 50 (4), 483–492. 10.1016/j.joen.2024.01.002 38237659

[B46] Guiotto M. Raffoul W. Hart A. M. Riehle M. O. di Summa P. G. (2020). Human platelet lysate to substitute fetal bovine serum in hMSC expansion for translational applications: a systematic review. J. Transl. Med. 18 (1), 351. 10.1186/s12967-020-02489-4 32933520 PMC7493356

[B47] Habib H. A. Heeba G. H. Khalifa M. M. A. (2021). Effect of combined therapy of mesenchymal stem cells with GLP-1 receptor agonist, exenatide, on early-onset nephropathy induced in diabetic rats. Eur. J. Pharmacol. 892, 173721. 10.1016/j.ejphar.2020.173721 33159934

[B48] Han Z. Rao J. S. Ramesh S. Hergesell J. Namsrai B. E. Etheridge M. L. (2023). Model-guided design and optimization of CPA perfusion protocols for whole organ cryopreservation. Ann. Biomed. Eng. 51 (10), 2216–2228. 10.1007/s10439-023-03255-5 37351756 PMC10518287

[B49] Hansen S. B. Højgaard L. D. Kastrup J. Ekblond A. Follin B. Juhl M. (2022). Optimizing an immunomodulatory potency assay for mesenchymal stromal cell. Front. Immunol. 13, 1085312. 10.3389/fimmu.2022.1085312 36578497 PMC9791065

[B50] Harris D. T. (2014). Stem cell banking for regenerative and personalized medicine. Biomedicines 2 (1), 50–79. 10.3390/biomedicines2010050 28548060 PMC5423479

[B51] He Q. Ye Z. Zhou Y. Tan W. S. (2018). Comparative study of mesenchymal stem cells from rat bone marrow and adipose tissue. Turk J. Biol. Turk Biyol. Derg. 42, 477–489. 10.3906/biy-1802-52 30983864 PMC6451846

[B52] Heng B. C. (2009). Effect of rho-associated kinase (ROCK) inhibitor Y-27632 on the post-thaw viability of cryopreserved human bone marrow-derived mesenchymal stem cells. Tissue Cell 41 (5), 376–380. 10.1016/j.tice.2009.01.004 19261317

[B53] Heydarzadeh S. Kheradmand Kia S. Boroomand S. Hedayati M. (2022). Recent developments in cell shipping methods. Biotechnol. Bioeng. 119 (11), 2985–3006. 10.1002/bit.28197 35898166

[B54] Hoogduijn M. J. de Witte S. F. H. Luk F. van den Hout-van Vroonhoven MCGN Ignatowicz L. Catar R. (2016). Effects of freeze-thawing and intravenous infusion on mesenchymal stromal cell gene expression. Stem Cells Dev. 25 (8), 586–597. 10.1089/scd.2015.0329 26914168

[B55] Huang P. Zhang C. Delawary M. Korchak J. A. Suda K. Zubair A. C. (2022). Development and evaluation of IL-6 overexpressing mesenchymal stem cells (MSCs). J. Tissue Eng. Regen. Med. 16 (3), 244–253. 10.1002/term.3274 34890489

[B56] ICHGCP (2025). First cell-based gene therapy for adult patients with relased or refractory MCL. Available online at: https://ichgcp.net/zh/news/fda-approves-first-cell-based-gene-therapy-for-adult-patients-with-relapsed-or-refractory-mcl. (Accessed December 16, 2025).

[B57] Jang T. H. Park S. C. Yang J. H. Kim J. Y. Seok J. H. Park U. S. (2017). Cryopreservation and its clinical applications. Integr. Med. Res. 6 (1), 12–18. 10.1016/j.imr.2016.12.001 28462139 PMC5395684

[B58] Jomha N. M. Elliott J. A. W. Law G. K. Maghdoori B. Forbes J. F. Abazari A. (2012). Vitrification of intact human articular cartilage. Biomaterials 33 (26), 6061–6068. 10.1016/j.biomaterials.2012.05.007 22698720

[B59] Kamprom W. Tangporncharoen R. Vongthaiwan N. Tragoonlugkana P. Phetfong J. Pruksapong C. (2024). Enhanced potent immunosuppression of intracellular adipose tissue-derived stem cell extract by priming with three-dimensional spheroid formation. Sci. Rep. 14 (1), 9084. 10.1038/s41598-024-59910-x 38643332 PMC11032398

[B60] Kanazawa S. Okada H. Riu D. Mabuchi Y. Akazawa C. Iwata J. (2022). Hematopoietic-mesenchymal signals regulate the properties of mesenchymal stem cells. Int. J. Mol. Sci. 23 (15), 8238. 10.3390/ijms23158238 35897814 PMC9330127

[B61] Kaushal R. Jahan S. McGregor C. Pineault N. (2022). Dimethyl sulfoxide-free cryopreservation solutions for hematopoietic stem cell grafts. Cytotherapy 24 (3), 272–281. 10.1016/j.jcyt.2021.09.002 34654640

[B62] Koung N. S. Shimizu M. Kaneda M. (2023). Characterization of rabbit mesenchymal stem/Stromal cells after cryopreservation. Biology 12 (10), 1312. 10.3390/biology12101312 37887022 PMC10603895

[B63] Kumar A. Ramesh S. Walther-Jallow L. Goos A. Kumar V. Ekblad Å. (2024). Successful transport across continents of GMP-manufactured and cryopreserved culture-expanded human fetal liver-derived mesenchymal stem cells for use in a clinical trial. Regen. Ther. 26, 324–333. 10.1016/j.reth.2024.06.012 39027723 PMC11255121

[B64] Kurtzberg J. Abdel-Azim H. Carpenter P. Chaudhury S. Horn B. Mahadeo K. (2020). A phase 3, Single-arm, prospective study of Remestemcel-L, *Ex Vivo* culture-expanded adult human mesenchymal stromal cells for the treatment of pediatric patients who failed to respond to steroid treatment for acute Graft-versus-Host disease. Biol. Blood Marrow Transpl. J. Am. Soc. Blood Marrow Transpl. 26 (5), 845–854. 10.1016/j.bbmt.2020.01.018 32018062 PMC8322819

[B65] Lam J. Lee B. Yu J. Kwee B. J. Kim Y. Kim J. (2022). A microphysiological system-based potency bioassay for the functional quality assessment of mesenchymal stromal cells targeting vasculogenesis. Biomaterials 290, 121826. 10.1016/j.biomaterials.2022.121826 36201944

[B66] Larsen K. Petrovski G. Boix-Lemonche G. (2024). Alternative cryoprotective agent for corneal stroma-derived mesenchymal stromal cells for clinical applications. Sci. Rep. 14 (1), 15788. 10.1038/s41598-024-65469-4 38982099 PMC11233711

[B67] Lawson A. Ahmad H. Sambanis A. (2011). Cytotoxicity effects of cryoprotectants as single-component and cocktail vitrification solutions. Cryobiology 62 (2), 115–122. 10.1016/j.cryobiol.2011.01.012 21262212 PMC3049861

[B68] Le Nail L. R. Brennan M. Rosset P. Deschaseaux F. Piloquet P. Pichon O. (2018). Comparison of Tumor- and bone marrow-derived mesenchymal stromal/stem cells from patients with high-grade osteosarcoma. Int. J. Mol. Sci. 19 (3), 707. 10.3390/ijms19030707 29494553 PMC5877568

[B69] Li X. Qian S. Song Y. Guo Y. Huang F. Han D. (2022). New insights into the mechanism of freeze-induced damage based on ice crystal morphology and exudate proteomics. Food Res. Int. Ott Ont. 161, 111757. 10.1016/j.foodres.2022.111757 36192927

[B70] Li H. Dai H. Li J. (2023). Immunomodulatory properties of mesenchymal stromal/stem cells: the link with metabolism. J. Adv. Res. 45, 15–29. 10.1016/j.jare.2022.05.012 35659923 PMC10006530

[B71] Li G. Zhao Y. Liu R. Zhang Y. Zhang Y. Du W. (2024). Highly effective strategy for isolation of mononuclear cells from frozen cord blood. J. Immunol. Methods 534, 113762. 10.1016/j.jim.2024.113762 39343085

[B72] Li X. Wei Y. Li W. Xu R. Xu C. Luo B. (2025). Acidic pH-Triggered membrane fusion enables lyophilized lipid nanoparticles as potent cryoprotectants for human erythrocytes. Adv. Healthc. Mater 3, e02455. 10.1002/adhm.202502455 40899629

[B73] Lin A. D. Y. Tung M. C. Lu C. H. (2021). The hernia sac-A suitable source for obtaining mesenchymal stem cells. Surg. Open Sci. 6, 40–44. 10.1016/j.sopen.2021.08.002 34632354 PMC8487083

[B74] Linkova D. D. Rubtsova Y. P. Egorikhina M. N. (2022). Cryostorage of mesenchymal stem cells and biomedical cell-based products. Cells 11 (17), 2691. 10.3390/cells11172691 36078098 PMC9454587

[B75] Liu L. Chen Y. Li X. Wang J. Yang L. (2024). Therapeutic potential: the role of mesenchymal stem cells from diverse sources and their derived exosomes in diabetic nephropathy. Biomed. Pharmacother. Biomedecine Pharmacother. 175, 116672. 10.1016/j.biopha.2024.116672 38677249

[B76] Luz-Crawford P. Kurte M. Bravo-Alegría J. Contreras R. Nova-Lamperti E. Tejedor G. (2013). Mesenchymal stem cells generate a CD4+CD25+Foxp3+ regulatory T cell population during the differentiation process of Th1 and Th17 cells. Stem Cell Res. Ther. 4 (3), 65. 10.1186/scrt216 23734780 PMC3706898

[B78] MacDonald E. S. Barrett J. G. (2019). The potential of mesenchymal stem cells to treat systemic inflammation in horses. Front. Vet. Sci. 6, 507. 10.3389/fvets.2019.00507 32039250 PMC6985200

[B79] Mallis P. Chatzistamatiou T. Dimou Z. Sarri E. F. Georgiou E. Salagianni M. (2022). Mesenchymal stromal cell delivery as a potential therapeutic strategy against COVID-19: promising evidence from *in vitro* results. World J. Biol. Chem. 13 (2), 47–65. 10.4331/wjbc.v13.i2.47 35432769 PMC8966500

[B80] Margiana R. Markov A. Zekiy A. O. Hamza M. U. Al-Dabbagh K. A. Al-Zubaidi S. H. (2022). Clinical application of mesenchymal stem cell in regenerative medicine: a narrative review. Stem Cell Res. Ther. 13 (1), 366. 10.1186/s13287-022-03054-0 35902958 PMC9330677

[B81] Martin K. E. Kalelkar P. P. Coronel M. M. Theriault H. S. Schneider R. S. García A. J. (2022). Host type 2 immune response to xenogeneic serum components impairs biomaterial-directed osteo-regenerative therapies. Biomaterials 286, 121601. 10.1016/j.biomaterials.2022.121601 35660823 PMC11458135

[B82] Martín-López M. Rosell-Valle C. Arribas-Arribas B. Fernández-Muñoz B. Jiménez R. Nogueras S. (2023). Bioengineered tissue and cell therapy products are efficiently cryopreserved with pathogen-inactivated human platelet lysate-based solutions. Stem Cell Res. Ther. 14 (1), 69. 10.1186/s13287-023-03300-z 37024935 PMC10079488

[B83] Medrano-Trochez C. Chatterjee P. Pradhan P. Stevens H. Y. Ogle M. E. Botchwey E. A. (2021). Single-cell RNA-seq of out-of-thaw mesenchymal stromal cells shows tissue-of-origin differences and inter-donor cell-cycle variations. Stem Cell Res. Ther. 12 (1), 565. 10.1186/s13287-021-02627-9 34736534 PMC8567133

[B84] Mendt M. Daher M. Basar R. Shanley M. Kumar B. Wei Inng F. L. (2021). Metabolic reprogramming of GMP grade cord tissue derived mesenchymal stem cells enhances their suppressive potential in GVHD. Front. Immunol. 12, 631353. 10.3389/fimmu.2021.631353 34017325 PMC8130860

[B85] Miroslava J. Pavel S. Doris V. Peter B. Alzbeta F. Stanislav F. (2020). New cryopreservation technology of hMSCs: first preclinical results using DMSO-containing medium. Cryo Lett. 41 (1), 50–56. 33973985

[B86] Mohamed H. M. Sundar P. Ridwan N. A. A. Cheong A. J. Mohamad Salleh N. A. Sulaiman N. (2024). Optimisation of cryopreservation conditions, including storage duration and revival methods, for the viability of human primary cells. BMC Mol. Cell Biol. 25 (1), 20. 10.1186/s12860-024-00516-6 39350017 PMC11441136

[B87] Moll G. Geißler S. Catar R. Ignatowicz L. Hoogduijn M. J. Strunk D. (2016). Cryopreserved or fresh mesenchymal stromal cells: only a matter of taste or key to unleash the full clinical potential of MSC therapy? Adv. Exp. Med. Biol. 951, 77–98. 10.1007/978-3-319-45457-3_7 27837556

[B88] Moreno D. F. Cid J. (2019). Graft-versus-host disease. Med. Clin. Barc. 152 (1), 22–28. 10.1016/j.medcli.2018.07.012 30309668

[B89] Murray A. Kilbride P. Gibson M. I. (2024). Trehalose in cryopreservation. Applications, mechanisms and intracellular delivery opportunities. RSC Med. Chem. 15 (9), 2980–2995. 10.1039/d4md00174e 39309363 PMC11411628

[B90] Naji A. Eitoku M. Favier B. Deschaseaux F. Rouas-Freiss N. Suganuma N. (2019). Biological functions of mesenchymal stem cells and clinical implications. Cell Mol. Life Sci. CMLS 76 (17), 3323–3348. 10.1007/s00018-019-03125-1 31055643 PMC11105258

[B91] Nicotra T. Desnos A. Halimi J. Antonot H. Reppel L. Belmas T. (2020). Mesenchymal stem/stromal cell quality control: validation of mixed lymphocyte reaction assay using flow cytometry according to ICH Q2(R1). Stem Cell Res. Ther. 11 (1), 426. 10.1186/s13287-020-01947-6 33004063 PMC7531151

[B92] Nishigaki T. Teramura Y. Nasu A. Takada K. Toguchida J. Iwata H. (2011). Highly efficient cryopreservation of human induced pluripotent stem cells using a dimethyl sulfoxide-free solution. Int. J. Dev. Biol. 55 (3), 305–311. 10.1387/ijdb.103145tn 21710436

[B93] Ntege E. H. Sunami H. Denda J. Futenma N. Shimizu Y. (2020). Effects of hydroxyapatite-coated nonwoven polyethylene/polypropylene fabric on non-mesodermal lineage-specific differentiation of human adipose-derived stem cells. BMC Res. Notes 13 (1), 471. 10.1186/s13104-020-05315-8 33028399 PMC7542906

[B94] Ortiz Silva N. A. Denis S. Vergnaud J. Hillaireau H. (2024). Controlled hydrogel-based encapsulation of macrophages determines cell survival and functionality upon cryopreservation. Int. J. Pharm. 650, 123491. 10.1016/j.ijpharm.2023.123491 37806508

[B95] Patel T. Mešić J. Meretzki S. Bronshtein T. Brlek P. Kivity V. (2025). Therapeutic potential and mechanisms of mesenchymal stem cells in coronary artery disease: narrative review. Int. J. Mol. Sci. 26 (11), 5414. 10.3390/ijms26115414 40508220 PMC12156323

[B96] Pilbauerova N. Schmidt J. Soukup T. Prat T. Nesporova K. Velebny V. (2022). Innovative approach in the cryogenic freezing medium for mesenchymal stem cells. Biomolecules 12 (5), 610. 10.3390/biom12050610 35625538 PMC9138570

[B97] Pola-Silva L. Xerfan Nahas F. Nascimento F. Santos T. R. Malinverni A. M. Alves A. (2021). Technique for obtaining mesenchymal stem cell from adipose tissue and stromal vascular fraction characterization in long-term cryopreservation. J. Vis. Exp. JoVE 30 (178). 10.3791/63036 35037654

[B98] Polchow B. Kebbel K. Schmiedeknecht G. Reichardt A. Henrich W. Hetzer R. (2012). Cryopreservation of human vascular umbilical cord cells under good manufacturing practice conditions for future cell banks. J. Transl. Med. 10, 98. 10.1186/1479-5876-10-98 22591741 PMC3422199

[B99] Pollock K. Sumstad D. Kadidlo D. McKenna D. H. Hubel A. (2015). Clinical mesenchymal stromal cell products undergo functional changes in response to freezing. Cytotherapy 17 (1), 38–45. 10.1016/j.jcyt.2014.06.008 25457275 PMC4274232

[B100] Prickett R. C. Marquez-Curtis L. A. Elliott J. A. W. McGann L. E. (2015). Effect of supercooling and cell volume on intracellular ice formation. Cryobiology 70 (2), 156–163. 10.1016/j.cryobiol.2015.02.002 25707695

[B101] Pu Z. Zhang L. Yang H. Shao T. Wang D. Wang J. (2025). Vitrification of 3D-MSCs encapsulated in GelMA hydrogel: improved cryosurvival, reduced cryoprotectant concentration, and enhanced wound healing. Int. J. Biol. Macromol. 296, 139716. 10.1016/j.ijbiomac.2025.139716 39809395

[B102] Putra I. Shen X. Anwar K. N. Rabiee B. Samaeekia R. Almazyad E. (2021). Preclinical evaluation of the safety and efficacy of cryopreserved bone marrow mesenchymal stromal cells for corneal repair. Transl. Vis. Sci. Technol. 10 (10), 3. 10.1167/tvst.10.10.3 34383879 PMC8362636

[B103] Rall W. F. Fahy G. M. (1985). Ice-free cryopreservation of mouse embryos at -196 degrees C by vitrification. Nature 313 (6003), 573–575. 10.1038/313573a0 3969158

[B104] Ramireddy A. Brodt C. R. Mendizabal A. M. DiFede D. L. Healy C. Goyal V. (2017). Effects of transendocardial stem cell injection on ventricular proarrhythmia in patients with ischemic cardiomyopathy: results from the POSEIDON and TAC-HFT trials. Stem Cells Transl. Med. 6 (5), 1366–1372. 10.1002/sctm.16-0328 28252842 PMC5442721

[B105] Rosell-Valle C. Antúnez C. Campos F. Gallot N. García-Arranz M. García-Olmo D. (2021). Evaluation of the effectiveness of a new cryopreservation system based on a two-compartment vial for the cryopreservation of cell therapy products. Cytotherapy 23 (8), 740–753. 10.1016/j.jcyt.2020.12.004 33714705

[B106] Rui K. Zhang Z. Tian J. Lin X. Wang X. Ma J. (2016). Olfactory ecto-mesenchymal stem cells possess immunoregulatory function and suppress autoimmune arthritis. Cell Mol. Immunol. 13 (3), 401–408. 10.1038/cmi.2015.82 26388237 PMC4856806

[B107] Rusconi G. Cremona M. Gallazzi M. Mariotta L. Gola M. Gandolfi E. (2024). Good manufacturing practice-compliant cryopreserved and thawed native adipose tissue ready for fat grafting. J. Clin. Med. 13 (11), 3028. 10.3390/jcm13113028 38892739 PMC11172459

[B108] Santos L. M. Shimabuko D. Y. Sipert C. R. (2024). Dimethyl sulfoxide affects the viability and mineralization activity of apical papilla cells *in vitro* . Braz Dent. J. 35, e246054. 10.1590/0103-644020246054 39699497 PMC11654018

[B77] Safwan M. Bourgleh M. S. Haider H. (2025). Clinical experience with cryopreserved mesenchymal stem cells for cardiovascular applications: a systematic review. World J. Stem Cells 17 (3), 102067. 10.4252/wjsc.v17.i3.102067 40160690 PMC11947892

[B109] Seo M. S. Kang K. K. Oh S. K. Sung S. E. Kim K. S. Kwon Y. S. (2021). Isolation and characterization of feline wharton’s jelly-derived mesenchymal stem cells. Vet. Sci. 8 (2), 24. 10.3390/vetsci8020024 33562192 PMC7915203

[B110] Sharpe P. T. (2016). Dental mesenchymal stem cells. Dev. Camb Engl. 143 (13), 2273–2280. 10.1242/dev.134189 27381225

[B111] Shi Y. Wang Y. Li Q. Liu K. Hou J. Shao C. (2018). Immunoregulatory mechanisms of mesenchymal stem and stromal cells in inflammatory diseases. Nat. Rev. Nephrol. 14 (8), 493–507. 10.1038/s41581-018-0023-5 29895977

[B112] Spees J. L. Lee R. H. Gregory C. A. (2016). Mechanisms of mesenchymal stem/stromal cell function. Stem Cell Res. Ther. 7 (1), 125. 10.1186/s13287-016-0363-7 27581859 PMC5007684

[B113] Stewart M. P. Langer R. Jensen K. F. (2018). Intracellular delivery by membrane disruption: mechanisms, strategies, and concepts. Chem. Rev. 118 (16), 7409–7531. 10.1021/acs.chemrev.7b00678 30052023 PMC6763210

[B114] Strecanska M. Sekelova T. Smolinska V. Kuniakova M. Nicodemou A. (2025). Automated manufacturing processes and platforms for large-scale production of clinical-grade mesenchymal Stem/stromal cells. Stem Cell Rev. Rep. 21 (2), 372–389. 10.1007/s12015-024-10812-5 39546186 PMC11872983

[B115] Svoradová A. Vašíček J. Zmrhal V. Venusová E. Pavlík A. Bauer M. (2023). Mesenchymal stem cells of Oravka chicken breed: promising path to biodiversity conservation. Poult. Sci. 102 (8), 102807. 10.1016/j.psj.2023.102807 37302325 PMC10276279

[B116] Swamynathan P. Venugopal P. Kannan S. Thej C. Kolkundar U. Bhagwat S. (2014). Are serum-free and xeno-free culture conditions ideal for large scale clinical grade expansion of Wharton’s jelly derived mesenchymal stem cells? A comparative study. Stem Cell Res. Ther. 5 (4), 88. 10.1186/scrt477 25069491 PMC4247668

[B117] Tan Y. Salkhordeh M. Wang J. P. McRae A. Souza-Moreira L. McIntyre L. (2019). Thawed mesenchymal stem cell product shows comparable immunomodulatory potency to cultured cells *in vitro* and in polymicrobial septic animals. Sci. Rep. 9 (1), 18078. 10.1038/s41598-019-54462-x 31792313 PMC6889371

[B118] Uberti B. Plaza A. Henríquez C. (2022). Pre-conditioning strategies for mesenchymal stromal/stem cells in inflammatory conditions of livestock species. Front. Vet. Sci. 9, 806069. 10.3389/fvets.2022.806069 35372550 PMC8974404

[B119] Vajta G. Holm P. Kuwayama M. Booth P. J. Jacobsen H. Greve T. (1998). Open pulled Straw (OPS) vitrification: a new way to reduce cryoinjuries of bovine ova and embryos. Mol. Reprod. Dev. 51 (1), 53–58. 10.1002/(SICI)1098-2795(199809)51:1<53::AID-MRD6>3.0.CO;2-V 9712317

[B120] Vymetalova L. Kucirkova T. Knopfova L. Pospisilova V. Kasko T. Lejdarova H. (2020). Large-Scale automated hollow-fiber bioreactor expansion of umbilical cord-derived human mesenchymal stromal cells for neurological disorders. Neurochem. Res. 45 (1), 204–214. 10.1007/s11064-019-02925-y 31828497

[B121] Wang H. Y. Lun Z. R. Lu S. S. (2011). Cryopreservation of umbilical cord blood-derived mesenchymal stem cells without dimethyl sulfoxide. Cryo Lett. 32 (1), 81–88. 21468457

[B122] Wang Y. Chen X. Cao W. Shi Y. (2014). Plasticity of mesenchymal stem cells in immunomodulation: pathological and therapeutic implications. Nat. Immunol. 15 (11), 1009–1016. 10.1038/ni.3002 25329189

[B123] Wang J. Zhao G. Zhang Z. Xu X. He X. (2016). Magnetic induction heating of superparamagnetic nanoparticles during rewarming augments the recovery of hUCM-MSCs cryopreserved by vitrification. Acta Biomater. 33, 264–274. 10.1016/j.actbio.2016.01.026 26802443 PMC5500173

[B124] Wang J. Shi X. Xiong M. Tan W. S. Cai H. (2022a). Trehalose glycopolymers for cryopreservation of tissue-engineered constructs. Cryobiology 104, 47–55. 10.1016/j.cryobiol.2021.11.004 34800528

[B125] Wang W. Yan M. Aarabi G. Peters U. Freytag M. Gosau M. (2022). Cultivation of cryopreserved human dental pulp stem Cells-A new approach to maintaining dental pulp tissue. Int. J. Mol. Sci. 23 (19), 11485. 10.3390/ijms231911485 36232787 PMC9570360

[B126] Wang X. Wang E. Zhao G. (2023). Advanced cryopreservation engineering strategies: the critical step to utilize stem cell products. Cell Regen. Lond Engl. 12 (1), 28. 10.1186/s13619-023-00173-8 37528321 PMC10393932

[B127] Weng L. (2023). Cell therapy drug product development: technical considerations and challenges. J. Pharm. Sci. 112 (10), 2615–2620. 10.1016/j.xphs.2023.08.001 37549846

[B128] Whaley D. Damyar K. Witek R. P. Mendoza A. Alexander M. Lakey J. R. (2021). Cryopreservation: an overview of principles and cell-specific considerations. Cell Transpl. 30, 963689721999617. 10.1177/0963689721999617 33757335 PMC7995302

[B129] Wiese D. M. Braid L. R. (2020). Transcriptome profiles acquired during cell expansion and licensing validate mesenchymal stromal cell lineage genes. Stem Cell Res. Ther. 11 (1), 357. 10.1186/s13287-020-01873-7 32795342 PMC7427746

[B130] Wiese D. M. Wood C. A. Braid L. R. (2022). From vial to vein: crucial gaps in mesenchymal stromal cell clinical trial reporting. Front. Cell Dev. Biol. 10, 867426. 10.3389/fcell.2022.867426 35493074 PMC9043315

[B131] Windrum P. Morris T. C. M. Drake M. B. Niederwieser D. Ruutu T. EBMT Chronic Leukaemia Working Party Complications Subcommittee (2005). EBMT chronic leukaemia working party complications subcommittee. Variation in dimethyl sulfoxide use in stem cell transplantation: a survey of EBMT centres. Bone Marrow Transpl. 36 (7), 601–603. 10.1038/sj.bmt.1705100 16044141

[B132] Wu M. Han Z. B. Liu J. F. Wang Y. W. Zhang J. Z. Li C. T. (2014). Serum-free media and the immunoregulatory properties of mesenchymal stem cells *in vivo* and *in vitro* . Cell Physiol. Biochem. Int. J. Exp. Cell Physiol. Biochem. Pharmacol. 33 (3), 569–580. 10.1159/000358635 24603109

[B133] Wu C. Mi Y. Song J. Zhang M. Wang C. (2025). The regulatory effect of human umbilical cord mesenchymal stem cells on the gut microbiota in diabetic nephropathy rats. Iran. J. Biotechnol. 23 (1), e3975. 10.30498/ijb.2025.472772.3975 40463945 PMC12128948

[B134] Yamatoya K. Nagai Y. Teramoto N. Kang W. Miyado K. Nakata K. (2023). Dimethyl sulfoxide-free cryopreservation of differentiated human neuronal cells. Biopreservation Biobanking 21 (6), 631–634. 10.1089/bio.2022.0180 36827090

[B135] Yang Y. Melzer C. Bucan V. von der Ohe J. Otte A. Hass R. (2016). Conditioned umbilical cord tissue provides a natural three-dimensional storage compartment as *in vitro* stem cell niche for human mesenchymal stroma/stem cells. Stem Cell Res. Ther. 7, 28. 10.1186/s13287-016-0289-0 26869167 PMC4751714

[B136] Yi J. Liang X. M. Zhao G. He X. (2014). An improved model for nucleation-limited ice formation in living cells during freezing. PloS One 9 (5), e98132. 10.1371/journal.pone.0098132 24852166 PMC4031135

[B137] Zanata F. Shaik S. Devireddy R. V. Wu X. Ferreira L. M. Gimble J. M. (2016). Cryopreserved adipose tissue-derived stromal/stem cells: potential for applications in clinic and therapy. Adv. Exp. Med. Biol. 951, 137–146. 10.1007/978-3-319-45457-3_11 27837560

[B138] Zhu X. Wang Y. Sun Z. Cheng W. Chen K. Gao X. (2025). Mesenchymal stem cells attenuate podocyte injury in diabetic nephropathy through the promotion of type 2 macrophage polarization. Stem Cells Dev. 34 (11–12), 258–270. 10.1089/scd.2025.0038 40417844

